# Fabrication and Physical Properties of Single-Crystalline Βeta-FeSi_2_ Nanowires

**DOI:** 10.1186/s11671-020-03425-7

**Published:** 2020-10-14

**Authors:** Chih-Yung Yang, Shu-Meng Yang, Yu-Yang Chen, Kuo-Chang Lu

**Affiliations:** 1 Department of Materials Science and Engineering, National Cheng Kung University, Tainan, 701 Taiwan; 2 Center for Micro/Nano Science and Technology, National Cheng Kung University, Tainan, 701 Taiwan

**Keywords:** β-FeSi_2_, Nanowire, CVD, Ferromagnetic, Field emission

## Abstract

In this study, self-catalyzed β-FeSi_2_ nanowires, having been wanted but seldom achieved in a furnace, were synthesized via chemical vapor deposition method where the fabrication of β-FeSi_2_ nanowires occurred on Si (100) substrates through the decomposition of the single-source precursor of anhydrous FeCl_3_ powders at 750–950 °C. We carefully varied temperatures, duration time, and the flow rates of carrier gases to control and investigate the growth of the nanowires. The morphology of the β-FeSi_2_ nanowires was observed with scanning electron microscopy (SEM), while the structure of them was analyzed with X-ray diffraction (XRD) and transmission electron microscopy (TEM). The growth mechanism has been proposed and the physical properties of the iron disilicide nanowires were measured as well. In terms of the magnetization of β-FeSi_2_, nanowires were found to be different from bulk and thin film; additionally, longer β-FeSi_2_ nanowires possessed better magnetic properties, showing the room-temperature ferromagnetic behavior. Field emission measurements demonstrate that β-FeSi_2_ nanowires can be applied in field emitters.

## Introduction

As the dimension of CMOS devices is down to the nanoscale, metal silicide technology will be even more significant; the substrate of many photonics and microelectronics devices has been silicon. Transition-metal silicides have been studied extensively owing to their outstanding properties, including low resistivity, and great stability [1–5]. For instance, CrSi_2_, β-FeSi_2_, and MnSi are suitable as thermoelectric materials due to their narrow energy gap and great thermostability [6]; NiSi, CoSi_2_, and TiSi_2_ are frequently utilized as materials of the metal gate for decreasing the resistance [7].

With excellent properties, such as high compatibility and low defect density, one-dimensional nanostructures are promising for current and future microelectronic devices [8], drawing widespread attention not only from academic studies but also from industry applications [9]. For the past few years, growth kinetics of various metal silicide nanowires, including transition-metal silicides and rare-earth silicides, has been studied [10–14].

There are different phases for iron disilicides [15–19], among which, the unusual characteristics of β-FeSi_2_ is particularly fascinating. As previously reported, β-FeSi_2_ nanowires were demonstrated to have important applications in the field of communication [20]; unfortunately, over the many years, few have been able to successfully repeat the fabrication of β-FeSi_2_ nanowires with chemical vapor deposition. For β-FeSi_2_, the room temperature equilibrium phase, the potential applications in light emitters, and infrared detectors for silicon-based optoelectronics are attributed to its direct bandgap. In this work, we report direct growth and structural characterization of the single crystalline β-FeSi_2_ nanowires via a chemical vapor deposition method. The as-synthesized β-FeSi_2_ nanowires exhibited the room-temperature ferromagnetic behavior. Field emission measurements show that the β-FeSi_2_ nanowires are great field emission materials.

## Methods

In this study, we synthesized β-iron disilicide nanowires using chemical vapor deposition with anhydrous FeCl_3_ powder as a precursor, silicon (100) substrates, and Ar carrier gas but without any catalysts. Silicon substrates were cleaned with 3 %-buffered HF and put in the downstream zone of the furnace; anhydrous FeCl_3_ powder was placed in an alumina boat upstream from the substrates, the temperature range of which was 750 ~ 950 °C. We carefully varied temperatures, duration time, and the flow rates of carrier gases in order to realize the factors that influenced the growth of β-iron disilicide nanowires. We utilized scanning electron microscopy (SEM) to investigate the morphology of the β-FeSi_2_ nanowires; X-ray diffraction (XRD) and transmission electron microscopy (TEM) studies were conducted for structural identification. In addition, characteristics such as magnetism and field emission property were measured. The magnetic property measurements of the β-FeSi_2_ nanowires were conducted by the Superconducting Quantum Interference Device (SQUID) with the VSM option, while field emission property was measured by Kiethly-237.

## Results and Discussion

**Fig. 1:**
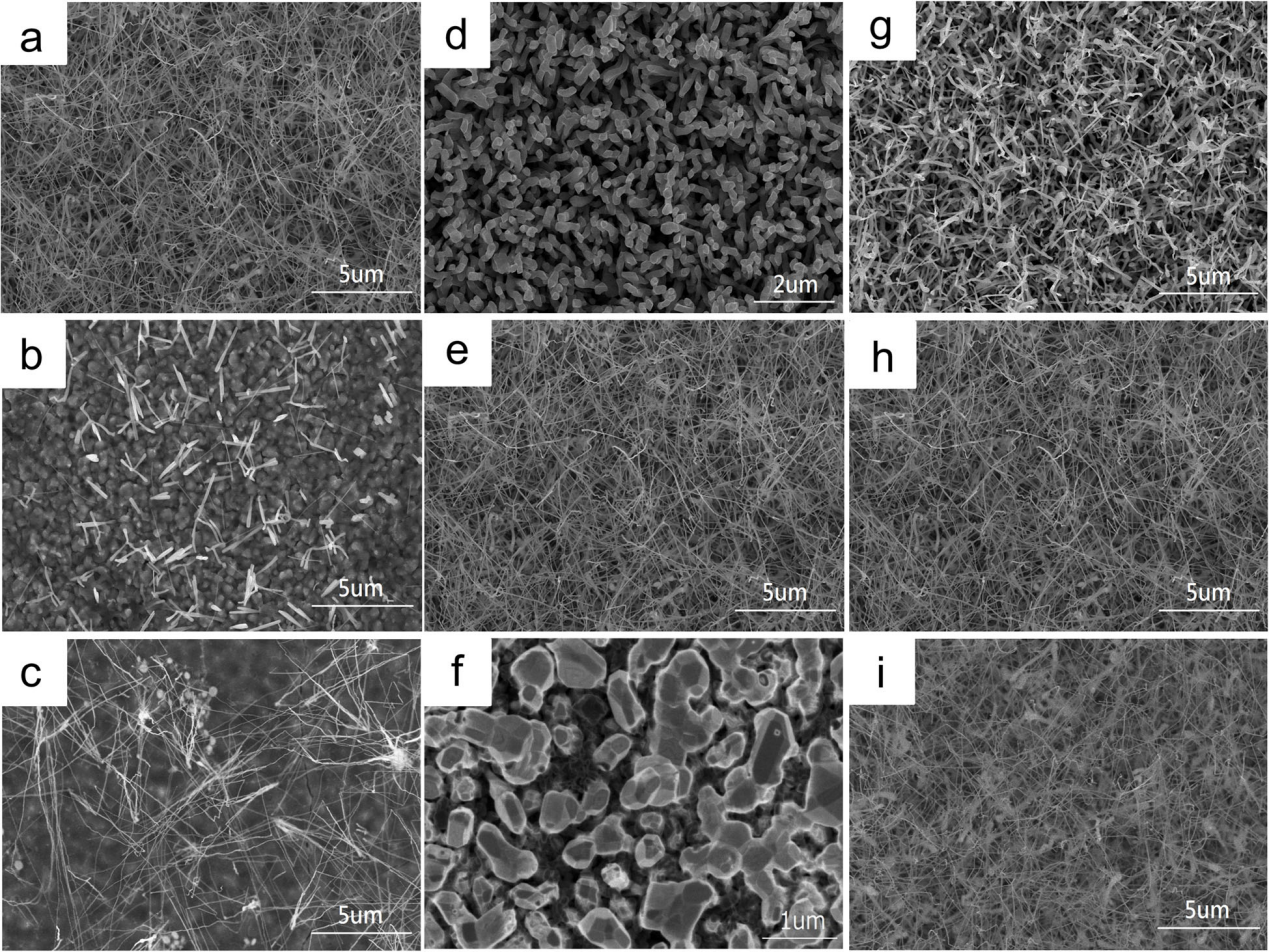
SEM images of β-FeSi_2_ nanowires at different parameters. At different gas flow rates: **a** 50 sccm, **b** 80 sccm, and **c** 120 sccm. At different temperatures: **d** 750 °C, **e** 850 °C, and **f** 950 °C. At different duration times: **g** 1 h, **h** 2 h, and **i** 5 h

We explored the parameters that could affect the growth of the β-iron disilicide nanowires. Firstly, different gas flow rates were investigated from 50 to 200 sccm as shown in the SEM images of Fig. 1a–c. Figure 1a reveals the gas flow rate at 50 sccm, where we found lots of nanowires with the diameters of 40 nm and lengths of 10 μm. Figure 1b shows the gas flow rate at 80 sccm, where there were some nanowires but the amount was reduced. In Fig. 1c showing the gas flow rate at 120 sccm, there were even fewer nanowires formed. According to the results, the amount of nanowires decreased with the increase of the gas flow rates. When the nanowires grow, the precursor, FeCl_3_, should be carried to the downstream zone of the tube furnace and react with the Si substrate by a carrier gas. At higher gas flow rates, it may be difficult for the nanowires to grow. Based on the chemical vapor deposition mechanisms, there were generally five steps in the deposition process, namely, (1) diffusion of reactants to the surface, (2) absorption of reactants at the surface, (3) chemical reaction at the surface, (4) desorption of products from the surface, and (5) diffusion of products from the surface. The slowest step determines the rate of the CVD reaction. If (1) or (5) is the slowest step, it is mass transfer-controlled. If (2), (3), or (4) is the slowest step, it can be called “surface reaction-controlled.” At low temperatures and slow gas flow rates, the surface chemical reaction is slower than reactant diffusion; thus, it is surface reaction-controlled. When it is surface reaction-controlled, variation in the film thickness across the wafer in the chamber will depend on the distribution of temperature, and thin film tends to form. However, our purpose is to grow nanowires; therefore, we should avoid surface reaction-controlled reaction. On the other hand, it is mass transfer-controlled at high temperatures and low gas flow rates. When it is mass transfer-controlled, the rate of the top obtained reactants is faster than that at the sidewall since the axial growth is faster than the radial growth; as a result, nanowires tend to form, and thus, we obtained many nanowires with decreasing gas flow rates. Therefore, mass transfer-controlled reaction is necessary for nanowire growth.

The second parameter we investigated was different growth temperatures as shown in the SEM images of Fig. 1d–f. Figure 1d reveals the growth temperature at 750 °C, where there were some nanowires but their lengths and diameters were short and small. Figure 1e shows the growth temperature at 850 °C, where we found lots of nanowires with the diameters of 40 nm and lengths of 10 μm. When we increased the growth temperature to 950 °C as shown in Fig. 1f, nanowires became nanorods due to more deposition of precursors. The third parameter we investigated was the duration time; Fig. 1g–i shows the SEM images for 1 h, 2 h, and 5 h. Generally, we found longer nanowires with the increasing duration time. After more than 5 h, the morphology of the nanowires would not change significantly, which may be attributed to the fact that the precursor had been completely consumed.

**Fig. 2:**
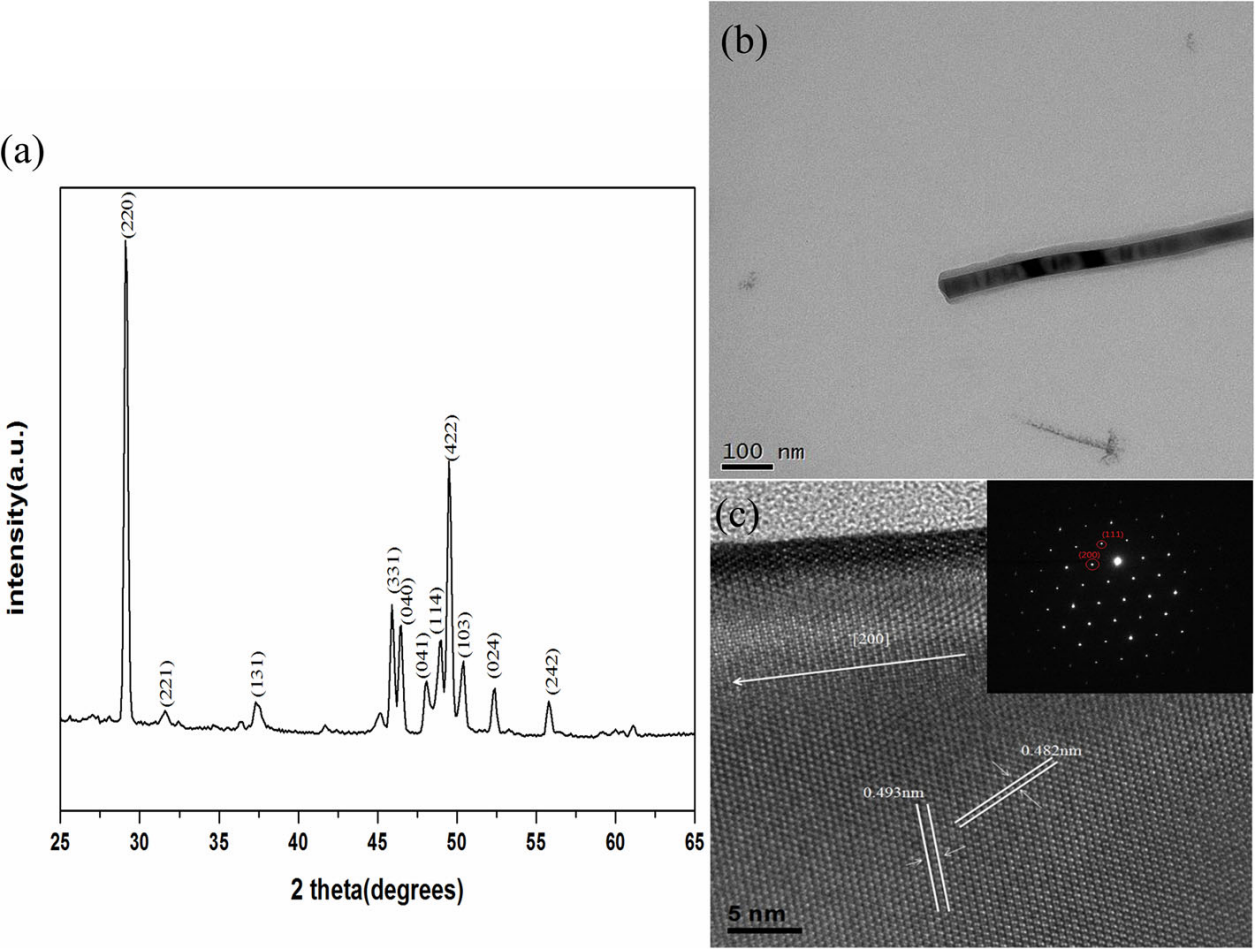
**a** XRD pattern for β-FeSi_2_ NWs, **b** a low-magnification TEM image of a β-FeSi_2_ NW nanowire, and **c** HRTEM of a β-FeSi_2_ NW. The inset in **c** is the corresponding diffraction pattern with [011] zone axis

To identify the structure of the nanowires, we conducted X-ray diffraction (XRD) and transmission electron microscopy (TEM) analysis as shown in Fig. 2. All the peaks in the corresponding XRD spectrum could be indexed to the structure of orthorhombic β-FeSi_2_ phase as shown in Fig. 2a. Figure 2b is a TEM image showing a single-crystalline β-FeSi_2_ nanowire; Fig. 2c is the high-resolution TEM image with the inset of the corresponding fast Fourier transform (FFT) diffraction pattern, showing that the β-FeSi_2_ nanowire has an orthorhombic structure with [200] growth direction and that the interplanar spacings of planes (200) and (111) are 0.493 nm and 0.482 nm, respectively.

**Fig. 3:**
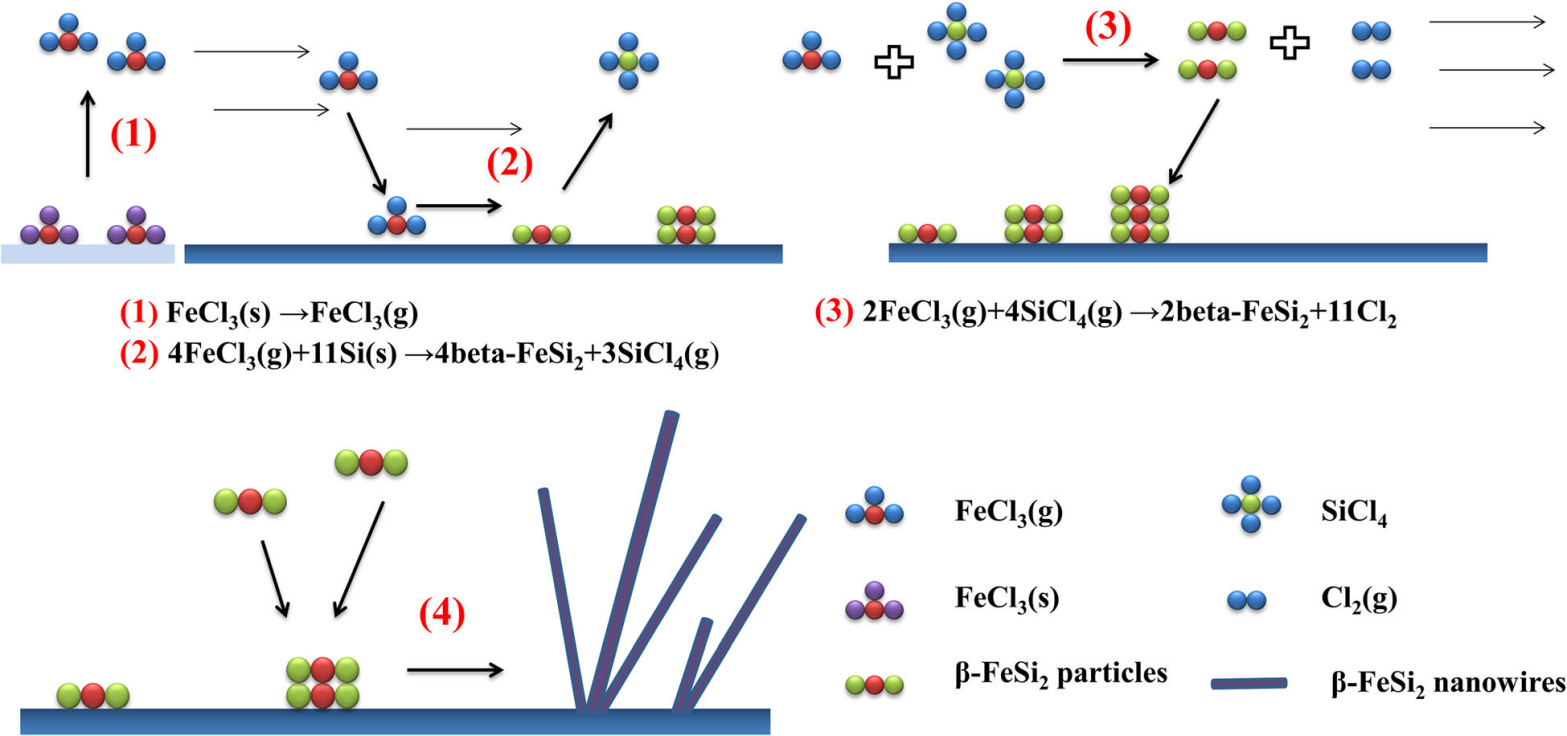
Schematic illustration of the growth mechanism **1** FeCl_3_(s) → FeCl_3_(g); **2** 4FeCl_3_(g) + 11Si(s) → 4β-FeSi_2_ + 3SiCl_4_(g); **3** 2FeCl_3_(g) + 4SiCl_4_(g) → 2β-FeSi_2_ + 11Cl_2_

The growth mechanism in our experiment may involve two reactions to produce β-FeSi_2_ nanowires as shown in Fig. 3. In the first reaction, evaporative FeCl_3_ was carried to the furnace downstream, reacting with the Si substrate to form β-FeSi_2_ nanoparticles and by-products of SiCl_4_ with β-FeSi_2_ nanoparticles appearing increasingly. In the second reaction, SiCl_4_ from the first reaction would also react with the precursor of evaporative FeCl_3_ and form β-FeSi_2_ and Cl_2_. With Cl_2_ carried out by Ar gas, we gradually obtained β-FeSi_2_ nanowires from both the first and second reactions. The growth mechanism was VS because we did not observe catalyst-like metal droplets at the front end of the nanowire. The synthesis via VLS mechanism requires a catalyst; however, no catalyst was used in the experiment. To further investigate the growth mechanism, we tried hydrogen, which may have a reduction effect; still, no metal catalytic droplet was formed. Therefore, we demonstrate that the growth mechanism was VS.

The magnetization of β-FeSi_2_ was interesting with different dimensions. It has been found to exhibit superparamagnetism in nanoparticles, even though no magnetic ordering occurs in bulk [21], while in the case of β-FeSi_2_ thin film, ferromagnetism was found only at temperatures below 100 K [22]. The ferromagnetic behavior of β-FeSi_2_ nanowires may be due to the large specific surface area of the nanowire, leading to many unpaired iron atoms on the surface. Additionally, some strain and defects generated during the growth process could be another factor contributing to the ferromagnetism. To examine the magnetic properties of the grown β-FeSi_2_ nanowires, the magnetic properties were measured using the Superconducting Quantum Interference Device (SQUID) with VSM option.

**Fig. 4:**
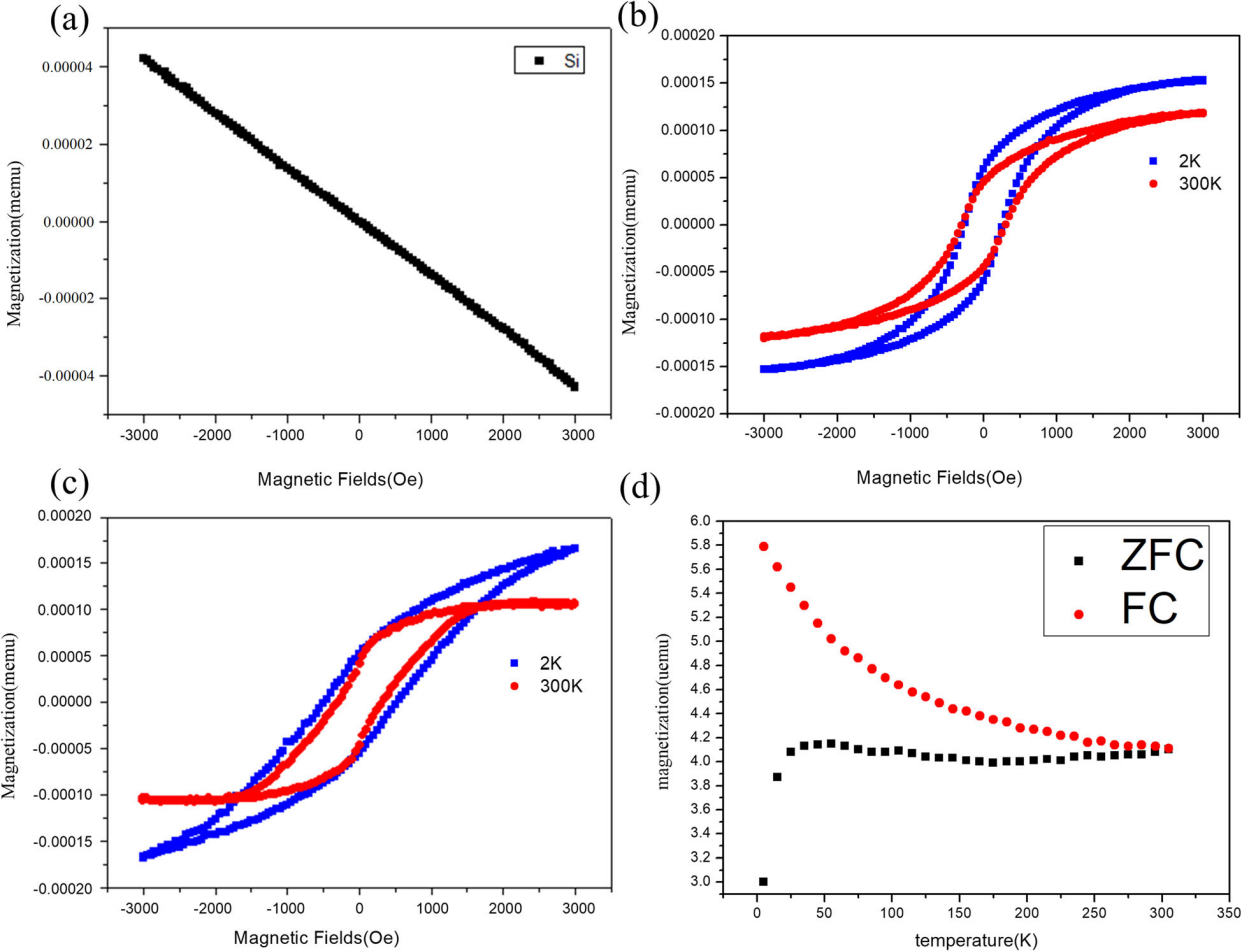
**a** Magnetization measurements of the Si substrate. **b** Magnetization measurements of the shorter β-FeSi_2_ nanowires at 2 K and 300 K. **c** Magnetization measurements of the longer β-FeSi_2_ nanowires at 2 K and 300 K. **d** Temperature-dependent magnetization of the β-FeSi_2_ nanowires

Figure 4a is the magnetic response only from the silicon substrate, which clearly shows diamagnetic behavior; we subtracted the magnetism of the silicon substrate for all the following magnetism of β-FeSi_2_ nanowires. The magnetization curve of the β-FeSi_2_ nanowires was growing in 2 h as shown in Fig. 4b. The nonlinear hysteresis loop curve shows that the β-FeSi_2_ nanowires exhibited ferromagnetic behavior at room temperature. The coercivity was about 264 Oe. Larger saturation magnetization was found at 2 K because of the decreasing thermal fluctuation. Due to the reduced coordination of the surface iron atoms, or the strain and structural defects in the crystal, β-FeSi_2_ nanowires grown here were found to be ferromagnetic [23]. Figure 4c shows the magnetization curve of the longer β-FeSi_2_ nanowires growing in 5 h. From shorter to longer nanowires, the coercivity increased from 264 to 345 Oe at 300 K, and even to 575 Oe at 2 K; saturation magnetization was raised more as well. It was confirmed that the longer nanowires possessed better magnetic properties. Temperature-dependent field cooling (FC) and zero-field cooling (ZFC) magnetization measurements are shown in Fig. 4d, where the magnetization curve did not drop to zero, showing that the curie temperature of β-FeSi_2_ NWs was higher than room temperature. The ZFC and FC curves of β-FeSi_2_ NWs did not overlap; the temperature of curve separation is called blocking temperature (T_b_), indicating that a large magnetic anisotropy energy barrier distribution existed [24]. When the temperature was lower than T_b_, the magnetic anisotropy energy was larger than the thermal fluctuation. As a result, grains were blocked and not impacted by the applied magnetic field; thus, the magnetization was observed.

**Fig. 5:**
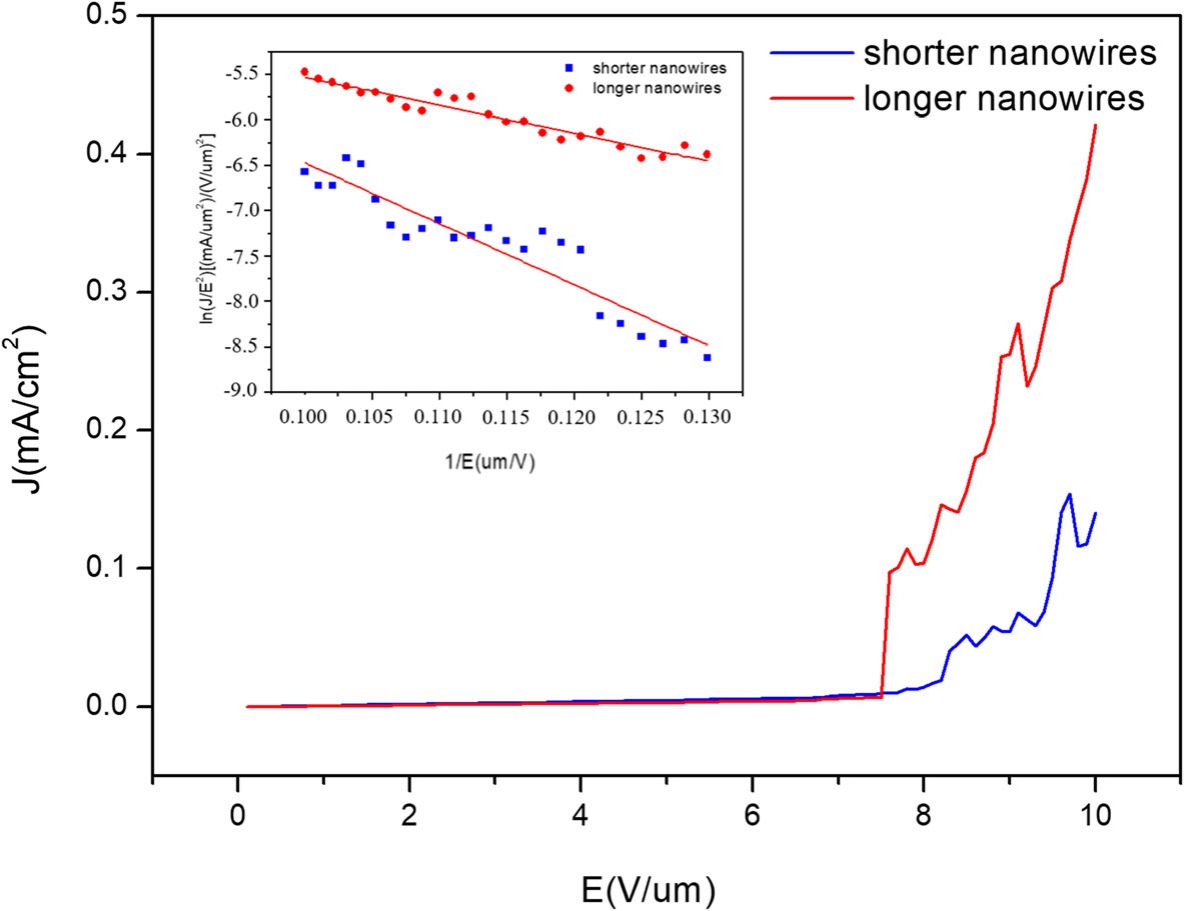
The field emission plot of β-FeSi_2_ NWs with different dimensions. The inset shows the corresponding ln(*J*/*E*^2^)-1/*E* plot

To explore the field emission properties, we conducted the field emission measurements for the β-FeSi_2_ nanowires. The sample was measured in a vacuum chamber at ~ 10^-6^ torr. Figure 5 shows the current density (*J*) - field (*E*) plot with β-FeSi_2_ nanowires of different lengths. According to the Fowler–Nordheim (F–N) plot and the Fowler–Nordheim equation:

$$ J=\left(\mathrm{A}{\ss}^2{E}^2/\varphi \right)\exp \left(-\mathrm{B}{\varphi}^{3/2}/\ss \mathrm{E}\right), $$

where *J* is the current density, *E* is the applied electric field, and *φ* is the work function; the inset reveals the ln(*J*/*E*^2^)-1/*E* plot. The field enhancement *ß* was calculated to be 1060 from the slope of ln(*J*/*E*^2^) = ln(A*ß*^2^/*φ*)-B*φ*^3/2^/*ßE*, and *ß* increased from 1060 to 2367 with the increasing length of nanowires, demonstrating that longer β-FeSi_2_ NWs had better field emission properties than shorter ones, and that β-FeSi_2_ NWs could be great field emission materials.

## Conclusions

β-FeSi_2_ nanowires were successfully synthesized with a CVD method. Processing parameters, including temperature, gas flow rate, and duration time were investigated for their effect on the nanowire growth. The growth mechanism has been proposed. Unlike bulk and thin-film β-FeSi_2,_ the as-synthesized β-FeSi_2_ nanowires exhibited room-temperature ferromagnetic behavior. Field emission measurements demonstrate the β-FeSi_2_ nanowires as potential field emitting materials.

## Data Availability

The data supporting our findings are included in the article.

## Abbreviations

SEM: Scanning electron microscopy
XRD: X-ray diffraction
TEM: Transmission electron microscopy
CMOS: Complementary metal-oxide-semiconductor
CVD: Chemical vapor deposition
FFT: Fast Fourier transform
HRTEM: High-resolution transmission electron microscope
VS: Vapor-solid method
VLS: Vapor–liquid–solid method
VSM: Vibrating sample magnetometer
SQUID: Superconducting quantum interference device
FC: Field cooling
ZFC: Zero-field cooling
T_b_: Blocking temperature
